# Complement Activation in 22q11.2 Deletion Syndrome

**DOI:** 10.1007/s10875-020-00766-x

**Published:** 2020-03-09

**Authors:** Dina Grinde, Torstein Øverland, Kari Lima, Camilla Schjalm, Tom Eirik Mollnes, Tore G. Abrahamsen

**Affiliations:** 1 Department of Pediatric Research, Oslo University Hospital, Oslo, Norway; 2 Department of Pediatric Medicine, Oslo University Hospital, Oslo, Norway; 3 Department of Endocrinology, Akershus University Hospital, Lørenskog, Norway; 4 Department of Immunology, Oslo University Hospital and University of Oslo, Oslo, Norway; 5 Research Laboratory, Nordland Hospital, Bodø, and K.G. Jebsen TREC, University of Tromsø, Tromsø, Norway; 6 Centre of Molecular Inflammation Research, Norwegian University of Science and Technology, Trondheim, Norway; 7 Center for Rare Diseases, Division of Pediatric and Adolescent Medicine, Oslo University Hospital, Oslo, Norway; 8 Faculty of Medicine, University of Oslo, Oslo, Norway

**Keywords:** 22q11.2del, 22q11.2 deletion syndrome, DiGeorge syndrome, primary immunodeficiencies, complement

## Abstract

The 22q11.2 deletion syndrome (22q11.2 del), also known as DiGeorge syndrome, is a genetic disorder with an estimated incidence of 1:3000 to 1:6000 births. These patients may suffer from affection of many organ systems with cardiac malformations, immunodeficiency, hypoparathyroidism, autoimmunity, palate anomalies, and psychiatric disorders being the most frequent. The importance of the complement system in 22q11.2 del has not been investigated. The objective of this study was to evaluate the complement system in relation to clinical and immunological parameters in patients. A national cohort of patients (*n* = 69) with a proven heterozygous deletion of chromosome 22q11.2 and a group of age and sex matched controls (*n* = 56) were studied. Functional capacity of the classical, lectin, and alternative pathways of the complement system as well as complement activation products C3bc and terminal complement complex (TCC) were accessed and correlated to clinical features. All patients in our study had normal complement activation in both classical and alternative pathways. The frequency of mannose-binding lectin deficiency was comparable to the normal population. The patients had significantly raised plasma levels of C3bc and a slight, but not significant, increase in TCC compared with controls. This increase was associated with the presence of psychiatric disorders in patients. The present study shows no complement deficiencies in 22q11.2 deletion syndrome. On the contrary, there are signs of increased complement activation in these patients. Complement activation is particularly associated with the presence of psychiatric disorders.

**Electronic supplementary material:**

The online version of this article (10.1007/s10875-020-00766-x) contains supplementary material, which is available to authorized users.

## Introduction

The 22q11.2 deletion syndrome (22q11.2 del), also known as DiGeorge or velocardiofacial syndrome, is the most common microdeletion syndrome in humans with an estimated prevalence from 1:3000 to 1: 6000 live births [1]. Due to clinical variability and heterogeneity, the occurrence of the disorder may be underestimated [2]. The prevalence is also expected to rise due to improved patient survival [1].

In 22q11.2 del, most of the patients have a typical 3 megabase (Mb) deletion on the long arm of chromosome 22 containing over 30 different genes. Approximately 8% of the patients have a smaller, 1.5 Mb, deletion and 2% have atypical deletions. The inheritance of the syndrome is autosomal dominant, but 90% of cases appear to be de novo [3, 4]. The phenotype variation is large, and syndrome presentation includes immunodeficiency of variable severity, congenital heart defects, hypoparathyroidism, velopharyngeal insufficiency (VPI), hypothyroidism, autoimmunity, obesity, variable cognitive delays, behavioral abnormalities, and psychiatric illnesses [1, 4, 5]. Indeed, over 180 clinical signs and symptoms have been described [6]. However, there is no apparent correlation between the size of the deletion and the clinical phenotype.

The complement system is an integral component of the innate immunity and can be activated through the classical, lectin, and alternative pathways. All three pathways are activated by different stimuli, and they converge on Complement 3 (C3), generating convertases that catalyze the conversion of C3 into its active fragments C3a and C3b [7]. This activation can be detected by different activation products of C3, including the C3b, iC3b, and C3c products (C3bc) exposing the same neoepitope in all fragments [8, 9]. C3b in the alternative pathway is the amplification step that leads to all downstream complement events in the cascade, which ends with the formation of the terminal C5b-9 complement complex (TCC) [7]. C5b-9 can be inserted into a membrane as the membrane attack complex and lyse bacteria and cells. It can be made in the fluid phase as soluble TCC and used as a marker indicating that the terminal pathway has been activated to its very end [8].

Complement activation is normally highly regulated. However, a disturbed complement activation can contribute to the pathology of several diseases associated with 22q11.2 del. Thus, complement activation is seen in autoimmune and inflammatory diseases such as systemic lupus erytomatosus, antiphospholipid antibody syndrome, or ANCA-associated vasculities [7]. Activation of the complement system has also been linked to obesity [10]. Moreover, dysregulation of complement has been demonstrated in both schizophrenia, bipolar disorders, and autism spectrum disorders (ASD), as involvement of the complement system in both synapse elimination and neuron migration in the developing brain has been recognized [11–17]. On the other hand, deficiency of complement components, including mannose-binding lectin (MBL), which is a part of lectin pathway, might increase the overall susceptibility to infectious disease [18].

The aim of the present study was to investigate the role of the complement system in the pathology of 22q11.2 del by studying the degree of complement activation in vivo and measure the level of MBL.

## Materials and Methods

### Patients

Sixty-nine patients, 32 males, median age 9 years (IQR 3.5–15 years) from all over Norway with a proven heterozygous deletion of chromosome 22 by fluorescent in situ hybridization or multiplex ligation-dependent probe amplification, were included in the study. They all attended the Pediatric Outpatient Clinic at Rikshospitalet, Oslo University Hospital, during the time period 2011–2015. Patients with atypical and known additional deletions were not included. The patients did not have any clinical apparent infection when sampled. Fifty-six healthy individuals, consisting of health care workers and their families or friends, 26 males, median age 10 years (IQR 5.3–19 years), were included as a control group. They had no known infection, inflammation, allergic disease, or other acute or chronic illness at the time of blood sampling. In some individuals, a restricted subset of tests was performed due to insufficient blood volume obtained. This is indicated in the figure legends.

The study was conducted according to the guidelines at our hospital and was approved by the Regional Committee for Research Ethics, reference number 2011/1741. Before inclusion, written informed consent was obtained from the participants and/or their parents.

### Blood Sampling

Peripheral venous blood was drawn into tubes containing EDTA. For plasma preparation, samples were stored on ice immediately after blood sampling and centrifuged at × 2000 for 15 min at 4 °C. All samples were aliquoted, stored at − 80 °C and thawed < 3 times.

### Clinical Chemistry and Immunology

Routine analysis including leukocyte differential count, immunoglobulins, hemoglobin, thrombocytes count, and C-reactive protein was performed at the Department of Medical Biochemistry, Oslo University Hospital. Lymphocyte subpopulation phenotyping was assayed at the Department of Immunology, Oslo University Hospital.

### Complement Analyses

Assay of functional capacity of the classical, lectin, and alternative pathways in the complement system was assayed in serum using Wielisa COMPL300 Total Complement Functional Screen kit from Wieslab AB, Lund, Sweden [19]. Classical pathway deficiency was defined as < 40% capacity. Lectin and alternative pathway deficiencies were defined as < 10% capacity.

Serum concentrations of MBL were quantified if lectin pathway deficiency was found using an MBL ELISA kit (BIOPORTO Diagnostics A/S, Hellerup, Denmark) according to the manufactures instructions. Low MBL value is most frequently set at < 500 ng/mL, and complete deficiency < 100 ng/mL.

In vivo complement activation was measured using activation products from C3 (C3bc) and the terminal pathway (TCC) using ELISA assays described in detail previously [20]. The C3bc assay is based on a capture monoclonal antibody (bH6) reacting with an epitope exposed in C3b, iC3b, and C3c. The TCC assay is based a capture monoclonal antibody (aE11) exposed in C9 when incorporated into TCC. Thus, both assays are highly specific for the activation products and not influenced by the amount of the native component.

### Weight

Trained nurses measured weight and height of the patients. Body mass index (BMI) was derived from height and weight based on the standard formula kg/m^2^. Adults were then classified as underweight (BMI < 18.5 kg/m^2^), normal weight (18.5 ≤ BMI < 25 kg/m^2^), overweight (25.0 ≤ BMI < 30.0 kg/m^2^), or obese (BMI ≥ 30.0 kg/m^2^) according to the World Health Organization criteria. For children and teens ages 2 through 19 years, normal weight was defined from 5th percentile to less than the 85th BMI-for-age percentile.

### Statistics

SPSS for Windows release 25 (Chicago, IL) was employed for the statistical analysis. For comparison of two groups, the non-parametric Mann-Whitney *U* test was used. When more than 2 groups of individuals were compared, the non-parametric Kruskall-Wallis test was used. If a significant difference was found, Mann-Whitney *U* test was used to calculate the difference between each pair of groups. Coefficients of correlation (*r*) were calculated by the non-parametric Spearman’s rank test. The strength of correlation was interpreted as previously described [21]. Categorical data were compared with a chi-squared test. Data are given as median and interquartile range (IQR) unless otherwise stated. Results were considered significant when *p* < 0.05. Figures were generated using GraphPad Prism version 7.04 for Windows (GraphPad Software, La Jolla, CA).

## Results

### Clinical Features of Patient Cohort

A total of 69 patients with a proven deletion of chromosome 22q11.2 were included in the analysis. Thirty-seven patients had congenital heart defects and 27 of those were of a conotruncal type. Nineteen patients had hypoparathyroidism and six patients had hypothyreosis at blood sampling. Nineteen patients had structural palate abnormalities, of whom 17 patients had cleft palate and two patients had a bifid uvula only. Fifteen patients had VPI diagnosis without having a cleft.

Two cases of hypothyroidism were of autoimmune type. Further, one patient had diabetes mellitus type I, and one was diagnosed with pernicious anemia and atrophic gastritis (parietal-cell and intrinsic factor autoantibodies positive). In addition, one patient suffered from sarcoidosis and one was diagnosed with Sjögren’s syndrome. Three patients had rosacea; two of those had another autoimmune or inflammatory disease mentioned above. Indeed, six out of seven patients above age of 30 years had symptoms of autoimmune or inflammatory disease.

Thirty-eight patients had normal weight and 16 patients were overweight or obese. None of the patients were underweight.

Forty-three patients had or have had psychiatric disorders such as neurodevelopmental disorders, including attention deficit hyperactivity disorder (ADHD), attention deficit disorder (ADD), ASD and intellectual disabilities (ID); neuropsychiatric disorders such as anxiety, major depressive disorder, and psychosis; learning disabilities and developmental delay. Six of those used psychotropic medications.

Thirty-one patients have or have had history of frequent sinopulmonary infections and otitis media. None of the patients had severe immunodeficiency. Immunological profile of patients is summarized in Supplementary Table 1.

All patients received standard treatment according to their additional diagnosis.

### Functional Capacity of Complement

In order to access a possible complement deficiency, we first studied classical, lectin, and alternative pathways by functional assays in 62 patients. All patients in our study had normal functional capacity of the classical and the alternative pathways. Five patients (8%) had pathologically low activation of lectin pathway; three of them (4.8%) had complete MBL deficiency with MBL levels < 100 ng/mL. In the general population, the prevalence of a congenital complement deficiency is rare and has been calculated to be about 0.03%, excluding MBL deficiency, estimated to occur in its homozygous form in about 5% of the population [22].

Since low lectin pathway activity nearly always is due to low MBL levels, we quantified MBL in these patients (Table 1). All five patients had low levels of MBL. Three of the patients had complete MBL deficiency (< 100 ng/ml) and the others had low MBL levels (< 500 ng/mL). Thus, all five low lectin pathway activities were explained by MBL deficiency. In our cohort, only one patient with MBL deficiency suffered from recurrent infection requiring hospital admission. This patient also had an IgA deficiency. Another patient had a history of recurrent otitis media. The three remaining patients have not had recurrent infections at all. Thus, despite the low number of patients, MBL deficiency was not associated with increased infections in this cohort, which is also consistent with the normal population where most individuals with MBL deficiency do not suffer from infections [23].

**Table 1:** Characteristics of 22q11.2 patients with MBL deficiency

	Demographics (sex, age)	Lectin pathway %	MBL μg/L	Increased infection susceptibility
Patient 1	F, 14 years	< 1	< 50	No
Patient 2	M, 5 years	2	130	No
Patient 3	F, 7 years	< 1	99	Recurrent laryngitis and LRTI^a^. IgA deficiency
Patient 4	39 years	< 1	< 50	No
Patient 5	12 years	3	178	No history of increased RTI^a^ History of recurrent otitis

^a^*LRTI* low respiratory tract infection, *RTI* respiratory tract infection

### Complement Activation

**Fig. 1:**
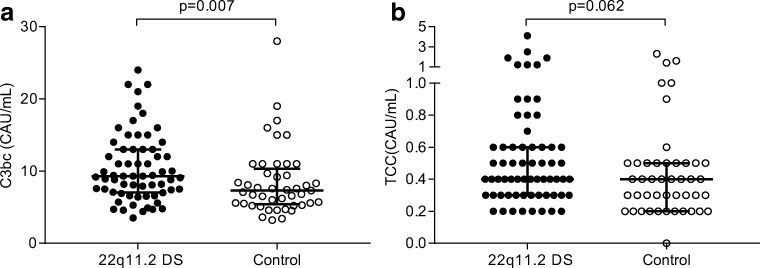
Analysis of complement activation products C3bc (**a**) and TCC (**b**) in 22q11.2 del patients (*n* = 64) and controls (*n* = 45). Individual symbols identify single subjects. Horizontal line represents median value and error bars represent interquartile range

In order to address if these patients had pathologic in vivo complement activation, we studied the complement activation products C3bc and TCC. The patients (*n* = 64) had significantly (*p* = 0.007) raised plasma levels of C3bc with a median value of 9.3 CAU/mL (IQR 7.0–13 CAU/mL) compared with controls (*n* = 45) where the corresponding values were 7.3 CAU/mL (IQR 5.4–10 CAU/mL) (Fig. 1a). Consistently, there was a slightly higher error bar level for level of TCC although the median levels did not differ and the total difference only showed a statistic trend (*p* = 0.62) (Fig. 1b). There was a weak, but significant correlation between C3bc and TCC in patients (*r* = 0.283, *p* = 0.024) and controls (*r* = 0.396, *p* = 0.007).

Further, we studied a possible correlation between age, graded in months, and plasma levels of C3bc and TCC and compared plasma levels of complement activation products between males and females in 22q11.2 del and in the control group. No correlation was found between age and plasma levels of C3bc (*r* = − 0.096, *p* = 0.530 in 22q11.2del and *r* = − 0.095, *p* = 0.455 in controls) or TCC (*r* = 0.048, *p* = 0.755 in 22q11.2del and *r* = 0.021, *p* = 0.868 in controls). No difference was found between levels of complement activation products with respect to sex neither in patients nor in controls (data not shown).

Since complement may modulate T cell response [7], we investigated correlation between plasma levels of C3bc and TCC and lymphocyte subpopulations in patients. We found weak, but significant negative correlation between C3bc and CD4+/CD45RA+ naïve T cells (*r* = − 0.350, *p* = 0.029). However, there was no significant correlation with CD4+/CD45RA+/CD31+ recent thymic emigrants (*r* = − 0.098, *p* = 0.552). As these two populations have a large degree of overlap, this discrepancy makes it difficult to interpret the first finding. Similarly, there was no correlation between the complement activation products and remaining lymphocyte subpopulations (Table S2).

### Relation of C3bc and TCC to Clinical Features

Because of the role of the complement system in the innate immune response, dysregulation of this system may be involved in the pathogenesis of 22q11.2 del. We therefore investigated if there was any association between plasma levels of C3bc, TCC, and clinical features of 22q11.2 del.

We did not find any difference in plasma levels of C3bc or TCC between 22q11.2 del patients with or without hypothyroidism, with or without history of autoimmune or inflammatory disease or of sinopulmonary infections, as well as between normal weight patients and obese or overweight patients (Table 2).

**Table 2:** Characteristics of 22q11.2 patients with MBL deficiency

Clinical features	C3bc		TCC	
	Value^a^	*p* value^b^	Value^a^	*p* value^b^
Hypoparathyroidism (*n* = 18)	10 (6.2–12)	0.881	0.5 (0.4–0.8)	0.204
Non-hypoparathyroidism (*n* = 46)	9.3 (7–13)		0.4 (0.3–0.6)	
Hypothyroidism (*n* = 5)	9.4 (4.9–13)	0.593	0.4 (0.2–0.5)	0.303
Non- hypothyroidism (*n* = 59)	9.3 (7.1–13)		0.4 (0.3–0.6)	
Autoimmune (*n* = 7)	8.8 (5.3–11)	0.302	0.4 (0.3–0.4)	0.209
Non-autoimmune (*n* = 57)	9.3 (7.2–13)		0.5 (0.3–0.7)	
Sinopulmonary infections (*n* = 29)	8.9 (6.5–13)	0.377	0.5 (0.3–0.7)	0.298
Non-sinopulmonary infections (*n* = 34)	9.6 (7.3–13)		0.4 (0.3–0.6)	
Normal weight (*n* = 36)	8.8 (6.7–12)	0.207	0.4 (0.3–0.6)	0.100
Overweight and obese (*n* = 15)	11 (7.9–16)		0.3 (0.2–0.5)	

^a^Values are expressed as CAU/mL, median (interquartile range)

^b^*p* values are calculated using Mann-Whitney *U* test

**Fig. 2:**
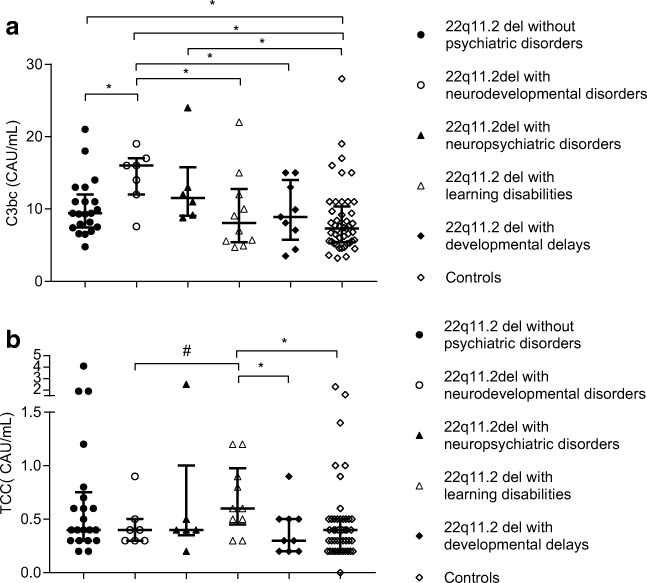
Plasma levels of C3bc (**a**) and TCC (**b**) in 22q11.2 del patients without psychiatric disorders (*n* = 21), and in patients with neurodevelopmental (*n* = 7) or neuropsychiatric (*n* = 6) disorders, learning disabilities (*n* = 10), developmental delays (*n* = 9), and controls (*n* = 45). Individual symbols identify single subjects. Horizontal line represents median value, error bars represent interquartile range. *p < 0.05, #*p* = 0.051

Patients with neurodevelopmental disorders (*n* = 7) had significantly raised plasma levels of C3bc with median value of 16 CAU/mL (IQR 12–17 CAU/mL) compared to patients without psychiatric disorders (*n* = 21, *p* = 0.019) where the corresponding values were 9.4 CAU/mL (IQR 7.4–12 CAU/mL), as well as to patients with learning difficulties (*n* = 10, *p* = 0.036) where the corresponding values were 8.0 CAU/mL (IQR 5.4–13 CAU/mL), patients with delayed development (*n* = 9, *p* = 0.030) were the corresponding values were 8.8 CAU/mL (IQR 5.8–14 CAU/mL) and healthy individuals (*p* = 0.002). Patients with neuropsychiatric disorders (*n* = 6) had significantly raised serum levels of C3bc with median value 12 CAU/mL (IQR 9–16 CAU/mL) compared to healthy individuals (*p* = 0.010). Patients without psychiatric disorders had significantly raised serum levels of C3bc (*p* = 0.018) compared to controls (Fig. 2a). Patients with learning disabilities had significantly raised plasma levels of TCC with median value of 0.57 CAU/mL (IQR 0.43–0.98 CAU/mL) compared to patients with developmental delay (*p* = 0.041) where the corresponding values were 0.35 CAU/mL (IQR 0.20–0.48 CAU/mL), as well as to healthy individuals (*n* = 45, *p* = 0.013) where the corresponding values were 0.38 CAU/mL (IQR 0.25–0.48 CAU/mL). Patients with neurodevelopmental disorders had lower plasma levels of TCC with median value of 0.36 CAU/mL (IQR 0.30–0.47 CAU/mL) compared to patients with learning disabilities (*p* = 0.051) (Fig. 2b). Patents using psychotropic drugs or receiving immunoglobulin supplementation (*n* = 7) were excluded from those analyses, as those medications are known to alter complement levels [24, 25].

## Discussion

In the present study, we investigated the complement system in patients with 22q11.2 del compared with controls. Our main finding was a significant increase in plasma levels of C3bc and a trend for increase in TCC in patients with 22q11.2 del compared to healthy individuals, suggesting an ongoing systemic in vivo complement activation. This increase was associated with the presence of psychiatric disorders in patients. Complement deficiencies was also screened for in the study, but no deficiency was detected, except for MBL deficiency, which was comparable to the normal population [26].

Management spectrum of immune deficiency in 22q11.2 del ranges from a thymus transplant to no intervention, due to the heterogeneity of the condition. Increased infection susceptibility in 22q11.2 del is due to immune deficiency, owing to thymic hypoplasia and impaired T cell production or unfavorable anatomy [1]. Because of the role of the complement system in the innate immune response, a possible additional complement deficiency in 22q11.2 del could also explain variation in infection severity and frequency. To our knowledge, complement deficiency in 22q11.2 del has not been investigated. In our study, we found no evidence of increased frequency of functional deficiency in classical or alternative pathways. Eight percent of 22q11.2 del patients had pathologically low activation of lectin pathway due to low MBL levels and 4.8% had complete MBL deficiency. However, variation in MBL concentration in apparently healthy individuals is large, and about one-third of the Caucasian population possess genotypes conferring low levels of MBL, with approximately 5% having very low levels [26]. Based on that, we conclude that the frequency of complement deficiency is definitely not increased in patients with 22q11.2 del and cannot explain any increased risk of infection.

In common variable immunodeficiency (CVID), Fevang et al. found no evidence of increased frequency of complement deficiencies [27]. However, the serum concentration of MBL was inversely correlated to the frequency of lower respiratory tract infections and bronchiectasis. In our cohort, one patient reported a previous history of recurrent otitis media, but not an increased infection rate. Further, one patient with MBL deficiency suffered from recurrent sinopulmonary infections requiring hospital admission. This patient also had an IgA deficiency, which is known to contribute to a more severe infection pattern [28]. Since three of five 22q11.2 del patients with MBL deficiency did not have any history of increased frequency or severity of infections, we suggest that a co-existing MBL deficiency does not contribute to infection severity in the 22q11.2 del.

Since complement activation due to the circulating immune complexes has been described in a variety of autoimmune diseases such as SLE, juvenile rheumatoid arthritis, and Sjögren’s syndrome [29], we hypothesized that increased complement activation seen in 22q11.2 del may be associated with the increased frequency of autoimmune diseases [30] seen in 22q11.2 del. We also hypothesized that increased complement activation may be associated with overweight seen in 22q11.2 del, since complement system has been linked to both obesity and metabolic syndrome [31]. Since we did not find any specific association between plasma levels of C3bc or TCC and autoimmunity, inflammation, hypothyroidism, or overweight, we speculate that increased complement activation is a general phenomenon in 22q11.2, reflecting unspecific pathophysiological processes going on in this disease. Complement is a general system reacting to any damaged self, irrespective of its origin, and the 22q11.2 del is a typical disease with possible exposure of a number of damaged self molecules recognized by complement, explaining the complement activation, which may propagate the disease processes.

The 22q11.2 del syndrome is associated with variable cognitive delays, learning disabilities, behavioral abnormalities and psychiatric illnesses such as ADHD, anxiety and schizophrenia [1]. In a large-scale international study on psychiatric disorders from childhood to adulthood in 22q11.2 del Schneider et al. demonstrated that ADHD is the most frequent disorder in childhood, affecting as many as 37% of patients [32]. Mood and anxiety disorders were common in 22q11.2 del and affected almost 31% of participants at all ages, but especially in children and adolescents. The frequency of major depressive disorder increased with age. Schizophrenia spectrum disorders were present in as many as 41% of adults over age 25, and early-onset psychosis was relatively common in individuals with 22q11.2 del, making 22q11.2 del the strongest known molecular genetic risk factor of schizophrenia [32]. Anxiety, obsessive-compulsive disorder, and depression in childhood have been identified as strong predictive factors for subsequent development of psychotic disorders in adulthood [33]. The high frequency of behavioral problems and psychiatric disorders in patients with 22q11.2 del suggests a very specific effect of the deletion [1]. The catechol-O-methyltransfease (COMPT) gene, which is responsible for metabolizing dopamine, is located within the commonly deleted region and it has been presumed that haploinsufficiency for COMPT contributes to the cognitive and behavioral findings [34].

In the present study, we found an association between increased plasma levels of C3bc, suggesting increased complement activation, and presence of psychiatric disorders in 22q11.2 del patients. TCC levels were increased in patients suffering from learning disabilities. This is of particular importance since several studies have shown involvement of the complement cascade in both brain development and brain disease [35]. In the healthy brain, complement proteins are expressed at relatively low levels and this expression vary with stages of maturation of neurons during neurodevelopment [14]. Neuronal migration is impaired in cases of ID, ASD, and schizophrenia, and complement pathway has been shown to be functionally important in migrating neurons of the developing cortex [12, 36]. Abnormal levels of complement proteins have been linked to ASD [37]. Sekar et al. discovered that schizophrenias association with variation MHC locus involves many common, structurally distinct C4 alleles that affect expression of C4A and C4B in the brain; each allele associated with schizophrenia risk in proportion to its effect on C4A expression [16]. Consistent with this, Fromer et al. found a strong correlation between the risk alleles for schizophrenia and upregulation of expression of C4A in post-mortem schizophrenia patients brains [38]. Indeed, 23 new studies on involvement of complement system in development of schizophrenia have been published in the time period 2008–2019, indicating that overall complement pathway activity appears to be elevated in schizophrenia [39]. Further, Reginia et al. demonstrated increased concentrations of C3a and C5a in the peripheral blood of patients suffering from bipolar disorder as compared to healthy individuals [15]. They also observed higher concentrations of C5b-9 in patients with bipolar disorder type II as compared to patients with bipolar disorder type I. Moreover, complement C3c and C4 were found to be significantly increased in unipolar depressed patients [40]. Thus, our findings are in conjunction with the observation of activation of the complement system in the pathogenesis of psychiatric disorders.

Main limitation of our study is that this is a cross-sectional study and psychiatric diagnoses were collected from the medical records. As 22q11.2 del typically presents with a wide range of psychiatric disorders, which may coexist or precede each other, classification of patients into different psychiatric subgroups is difficult. In DSM-5, specific learning disorders, motor disorders, communication disorders, ASD, ADHD, and ID are all placed in a neurodevelopmental cluster [41]. This group is heterogenic, and there is a number of differences between these disorders. In addition, neurodevelopmental impairment has been demonstrated in relation to schizophrenia, but schizophrenia is not classified as a neurodevelopmental disorder. Thus, ideally, those disorders should be analyzed separately. Another consideration to be made is the fact that in our study children and adolescents were over-represented, probably because the syndrome is still under-recognized in adults [42]. Some patients in the non-psychiatric group had high plasma levels of C3bc, and we cannot exclude that these children may develop psychiatric disorders later in life. In a longitudinal population-based study, Föcking et al. demonstrated that complement pathway changes at age 12 are associated with psychotic experiences at age 18 [43]. Therefore, there is a need for a follow-up longitudinal study to investigate the role of complement activation in psychiatric disorders in childhood and adulthood. This is also necessary because cognitive decline can precede the onset of psychiatric symptoms in 22q11.2 del [44]. Even that the number of patients included in our study is large for a 22q11.2 del syndrome cohort, it is also important to perform a larger study in order to access if there is any difference in complement activation between different psychiatric diagnoses seen in this syndrome.

Another limitation of our study is that, for C3bc, our control group contained far more individuals with levels above the reference range established by Bergseth et al. [20]. This difference can probably be explained by the inter-assay variation and highlights the importance of control group inclusion.

The knowledge about involvement of the complement system in pathophysiology of psychiatric disorders is relatively new and additional studies have been advocated [14]. In our opinion, the results from our study can contribute to the understanding of the pathophysiology of the psychiatric disorders in 22q11.2 del in general, but further studies are needed in order to understand the exact role of the immune system in psychiatric disorders. An important question is whether the complement activation seen is a result of the psychiatric illness itself or whether complement activation, somehow associated with 22q.11 del, acts as a co-factor in the development of the psychiatric illness.

## Electronic Supplementary Material

ESM 1 (PDF 217 kb)
