# Supplementary material for: Complement Activation in 22q11.2 Deletion Syndrome
Source: J Clin Immunol. 2020 Mar 9;40(3):515–23. doi: 10.1007/s10875-020-00766-x (PMC7142058; doi:10.1007/s10875-020-00766-x)
Supplement: Supplementary file 1 — (PDF 217 kb) [file 10875_2020_766_MOESM1_ESM.pdf]

Supplementary material

Complement Activation in 22q11.2 Deletion Syndrome

Journal of Clinical Immunology

Dina Grinde[1], Torstein Øverland[2], Kari Lima[2,3] , Camilla Schjalm[4], Tom Eirik Mollnes[4,5,6] and Tore G. Abrahamsen[7,8]

[1] Department of Pediatric Research, Oslo University Hospital, Oslo, Norway

[2] Department of Pediatric Medicine, Oslo University Hospital, Oslo, Norway

[3] Department of Endocrinology, Akershus University Hospital, Lørenskog, Norway

[4] Department of Immunology, Oslo University Hospital and University of Oslo, Norway

[5] Research Laboratory, Nordland Hospital, Bodø, and K.G. Jebsen TREC, University of Tromsø, Norway

[6] Centre of Molecular Inflammation Research, Norwegian University of Science and Technology, Trondheim, Norway

[7] Center for Rare Diseases, Division of Pediatric and Adolescent Medicine, Oslo University Hospital, Oslo, Norway

[8] Faculty of Medicine, University of Oslo, Oslo, Norway

Corresponding author:

Dina Grinde, Department of Pediatric Research, Oslo University Hospital, Oslo, Norway

E-mail: dina.aresvik@rr-research.no

Table S1 Immunological profile of patients with 22q11.2del

Parameters		Age groups						
		2-5 mo	5-9 mo	15-24 mo	2-5 yr	5-10 yr	10-16 yr	Adults
Lymphocyte subpopulations								
CD3+[b]	22q11.2 del[a]	1.6	2.2	1.8	1.4	1.3	1.1	1.1
	Ref.range[h]	3.4-12.2	1.8-18.7	1.4-12.1	1.4-5.5	1.2-4.7	1.4-4.2	1.2-4.1
CD4+[b]	22q11.2 del[a]	1.2	1.4	1.0	0.8	0.7	0.5	0.6
	Ref.range[h]	1.6-6.5	1.0-7.2	0.4-7.2	0.5-2.7	0.4-2.5	0.4-2.1	0.5-2.0
CD8+ T[b]	22q11.2 del[a]	0.3	0.7	0.5	0.5	0.4	0.5	0.4
	Ref.range[h]	0.3-3.4	0.2-5.4	0.2-2.8	0.2-1.8	0.2-1.7	0.3-1.3	0.2-1.2
CD19+[b]	22q11.2 del[a]	1.13	1.45	2.18	1.05	0.45	0.31	0.19

	Ref.range[h]	0.52-2.3	0.13-6.3	0.16-3.7	0.18-1.3	0.10-0.80	0.12-0.74	0.064-0.82
CD16+/CD56+/CD3- NK[b]	22q11.2 del[a]	0.976	0.775	0.412	0.410	0.040	0.363	0.249
	Ref.range[h]	0.097-1.99	0.068-3.9	0.055-4.0	0.061-0.51	0.070-0.59	0.092-1.2	0.10-1.2
CD3+/CD4-/CD8-/TCR alfa/beta + double negative[c]	22q11.2 del[a]	1.4	0.6	1.4	1.4	1.5	1.2	0.5
	Ref.range[h]	0.18-3	0.39-4	0.55-4	1-7	0.77-6	0.54-6	0.57-5
CD4+/CD45RO+ memory[d]	22q11.2 del[a]	25	29	33	38	45	63	50
	Ref.range[h]	2-51	2-59	0.09-40	15-56	0.035-100	13-76	18-95
CD4+/CD45RO+/CXCR5+ follicular-like[d]	22q11.2 del[a]	2	3	8	10	11	11	11
	Ref.range[h]	7-33	9-47	8-51	6-72	7-85	7-47	5-56
CD4+/CD45RA+ naïve[d]	22q11.2 del[a]	76	75	75	71	66	51	48
	Ref.range[h]	73 – 100	77 – 97	56 – 100	52 – 92	46 – 99	37-97	16-100
CD4+/CD45RA+/CD31+ recent thymic	22q11.2 del[a]	69	74	68	78	73	71	65

emigrants[e]	Ref.range[h]	64-94	65-90	40-100	37-100	41-81	31-81	7-100
CD4+/CD45RO+/CD127low/CD25+ regulatory[d]	22q11.2 del[a]	6	4	7	6	6	6	5
	Ref.range[h]	6-11	4-18	6-13	3-17	4-14	4-20	4-17
CD8+/CD27+/CD28+ naïve[f]	22q11.2 del[a]	95	43	84	72	66	54	58
	Ref.range[h]	47-100	31-100	10-100	19-100	16-100	20-95	6-100
CD8+/CD27+/CD28+ early effector/memory[f]	22q11.2 del[a]	5	15	12	19	12	9	10
	Ref.range[h]	0.08-24	0.1-13	1-8	1-9	1-6	0.42-18	1-20
CD8+/CD27-/CD28- late effector/memory[f]	22q11.2 del[a]	0.20	16	0.48	4	15	35	25
	Ref.range[h]	0.47-70	1-100	2-100	10-55	5-100	4-100	14-98

mo, month; yr, years

[a] Values are expressed as median

[b] Absolute counts ($x10^9$/L)

[c] % of CD3+ T lymphocytes

[d] % of CD4+ T lymphocytes

[e] % of CD4+/CD45RA+ T lymphocytes

[f] % of CD8+ T lymphocytes

[g] g/L

[h] Reference range as reported by Schatorjé et al [1]

Table S2 Correlations coefficients between C3bc, TCC and lymphocyte subpopulations in 22q11.2 deletion syndrome

Parameters	C3bc[a]		TCC[a]	
	r	p-value	r	p-value
Lymphocyte subpopulations (n=52)				
CD3+ T lymphocytes	-0.173	0.220	-0.059	0.677
CD4+ T lymphocytes	-0.184	0.192	-0.086	0.546
CD8+ T lymphocytes	-0.086	0.543	0.046	0.747
CD19+ B lymphocytes	0.029	0.838	-0.020	0.890
CD16+/CD56+/CD3- NK cells	0.009	0.950	-0.015	0.914
T lymphocytes subpopulations (n=39)				
CD3+/CD4-/CD8-/TCR alfa/beta + double negative	0.111	0.500	-0.098	0.553

CD4+/CD45RO+ memory	0.207	0.205	0.189	0.250
CD4+/CD45RO+/CXCR5+ follicular-like	0.146	0.374	-0.036	0.828
CD4+/CD45RA+ naïve	-0.350	0.029*	-0.071	0.668
CD4+/CD45RA+/CD31+ recent thymic emigrants	-0.098	0.552	-0.017	0.918
CD4+/CD45RO+/CD127low/CD25+ regulatory	-0.054	0.742	0.111	0.501
CD4+ naïve/memory ratio	-0.116	0.481	-0.094	0.569
CD8+/CD27+/CD28+ naïve	-0.087	0.601	-0.018	0.914
CD8+/CD27+/CD28+ early effector/memory	-0.243	0.141	-0.215	0.196
CD8+/CD27-/CD28- late effector/memory	0.180	0.280	0.105	0.530

[a] Data are present as Spearman's rho correlation coefficients with corresponding p-values

* $p < 0.05$

References

1. Schatorje EJ, Gemen EF, Driessen GJ, Leuvenink J, van Hout RW, de Vries E. Paediatric reference values for the peripheral T cell compartment. Scand J Immunol. 2012;75(4):436-44. doi:10.1111/j.1365-3083.2012.02671.x.
